# New treatment alternatives for primary and metastatic colorectal cancer by an integrated transcriptome and network analyses

**DOI:** 10.1038/s41598-024-59101-8

**Published:** 2024-04-16

**Authors:** Caner Karaca, Ezgi Demir Karaman, Asim Leblebici, Hasan Kurter, Hulya Ellidokuz, Altug Koc, Ender Berat Ellidokuz, Zerrin Isik, Yasemin Basbinar

**Affiliations:** 1 Department of Translational Oncology, Institute of Health Sciences, Dokuz Eylul University, Izmir, Turkey; 2 Department of Computer Engineering, Faculty of Engineering, Dokuz Eylul University, Izmir, Turkey; 3 Department of Preventive Oncology, Institute of Oncology, Dokuz Eylul University, Izmir, Turkey; 4 Department of Gastroenterology, Faculty of Medicine, Dokuz Eylul University, Izmir, Turkey; 5 Department of Translational Oncology, Institute of Oncology, Dokuz Eylul University, Izmir, Turkey

**Keywords:** Cancer genomics, Gastrointestinal cancer, Metastasis, Tumour biomarkers, Data integration, Functional clustering, Microarrays, Virtual drug screening

## Abstract

Metastatic colorectal cancer (CRC) is still in need of effective treatments. This study applies a holistic approach to propose new targets for treatment of primary and liver metastatic CRC and investigates their therapeutic potential in-vitro. An integrative analysis of primary and metastatic CRC samples was implemented for alternative target and treatment proposals. Integrated microarray samples were grouped based on a co-expression network analysis. Significant gene modules correlated with primary CRC and metastatic phenotypes were identified. Network clustering and pathway enrichments were applied to gene modules to prioritize potential targets, which were shortlisted by independent validation. Finally, drug-target interaction search led to three agents for primary and liver metastatic CRC phenotypes. Hesperadin and BAY-1217389 suppress colony formation over a 14-day period, with Hesperadin showing additional efficacy in reducing cell viability within 48 h. As both candidates target the G2/M phase proteins NEK2 or TTK, we confirmed their anti-proliferative properties by Ki-67 staining. Hesperadinin particular arrested the cell cycle at the G2/M phase. IL-29A treatment reduced migration and invasion capacities of TGF-β induced metastatic cell lines. In addition, this anti-metastatic treatment attenuated TGF-β dependent mesenchymal transition. Network analysis suggests IL-29A induces the JAK/STAT pathway in a preventive manner.

## Introduction

Metastasis is the major cause of death in patients with colorectal cancer^1^. The most common metastasis sites of colorectal cancer are the liver, followed by the lung, lymph node, and peritoneum^2^. Chemotherapy, immunotherapy, targeted therapy, and their combinations are used for metastatic colorectal cancer (CRC) treatment^3^. Even despite these current therapies, metastatic CRC still needs enhanced treatments.

Cell cycle regulators have emerged as prominent targets in cancer treatment, considering that cancer is fundamentally characterized by uncontrolled proliferation. Various chemotherapeutic agents aim to hinder the function of checkpoint proteins involved in cell division, particularly cyclin-dependent kinases (CDKs), to impede the signaling for cell division. FDA-approved examples are palbociclib, ribociclib, and abemaciclib, which specifically inhibit CDK 4/6. Similarly, the regulation of the G2/M transition and the proper separation of sister chromatids during mitosis have been targeted to obstruct cell division and induce aneuploidy. This approach is frequently employed, often in combination with other chemotherapy drugs, utilizing aurora kinase inhibitors and taxane-based chemotherapeutics in the treatment of various cancers.

The IL-10 family consists of a wide range of cytokines. IL-28A, IL-28B, and IL-29 form the subfamily of IL-20^4^. IL-29 exhibits multiple biological activities including immune regulatory activity, anti-viral^5^, obesity-induced inflammation^6^, and anti-tumor properties^7^. IL-29 has been shown to have anti-tumor effects on many human cancer cells such as melanoma^8^, neuroendocrine cancer^9^, colorectal cancer^10^, and glioblastoma^11^. However, there is no descriptive study on the effect of IL-29 on the metastatic phenotype of colorectal cancer. In this study, the anti-metastatic effect of IL-29 on colorectal cancer cell lines obtained from different metastatic sites was demonstrated for the first time.

Recent bioinformatics studies applied co-expression networks, differential expression gene (DEG), pathway, and protein–protein interaction (PPI) analysis on CRC samples. A study used weighted gene co-expression network analysis (WGCNA) on CRC samples obtained from two Gene Expression Omnibus (GEO) datasets^12^. After differentially expressed genes of important modules were mapped on the STRING network, then the genes with higher degrees were identified as hubs, which were associated with the overall survival of patients. A similar analysis workflow was applied, and three hub genes (HCLS1, EVI2B, and CD48) were validated as tumor suppressors by the qPCR method^13^. Another study identified significantly expressed genes between four CRC datasets^14^. The common genes were clustered on the STRING network; core genes of modules were analyzed in terms of survival effects and protein levels. A study considered samples of CRC with liver metastasis to identify metastasis-related pathways^15^. They applied DEG analysis and WGCNA, finally revealing the roles of the complement-coagulation cascade and the focal adhesion pathway in CRC progression. Commonly expressed genes of several CRC datasets revealed new biomarkers, which were validated by in-silico analysis to propose new therapeutic agents for CRC treatment^16^. Although several studies considered primary samples to reveal the main reasons for CRC development, meta data analysis is quite limited for understanding CRC with liver metastasis.

The current study reveals new targets for the treatment of primary CRC and liver metastatic ones. Microarray samples of CRC patients were integrated from different GEO datasets. The integrated samples were analyzed by WGCNA. Significant modules were identified, which are highly correlated with primary and metastatic phenotypes. The important modules were further analyzed by applying network clustering algorithms. The differential expression and pathway enrichment analysis revealed target genes, which were validated on two independent datasets. The potential compounds targeting selected biomarkers were found by considering gene expression regulations. As a final step, three candidate compounds were validated by in-vitro experiments to show their anti-proliferative effects on CRC cell lines.

## Results

This section presents the experimental results of the applied analysis. The computational results are followed by experimental validations.

### Gene co-expression analysis

The training dataset was analyzed using the WGCNA method to create a co-expression network and identify gene modules that were highly correlated with given phenotypes. WGCNA utilizes the scale-free topology criterion to construct a gene co-expression network^17^. The choice of the soft thresholding power (β) is crucial as it determines the co-expression similarity to calculate adjacency. To identify the best soft threshold, a network topology analysis was conducted for various values, as shown in Supplementary Fig. S1. The scale-free topology fit index curve's highest value was obtained before it flattened out, yielding the soft threshold value of "8".

**Figure 1:**
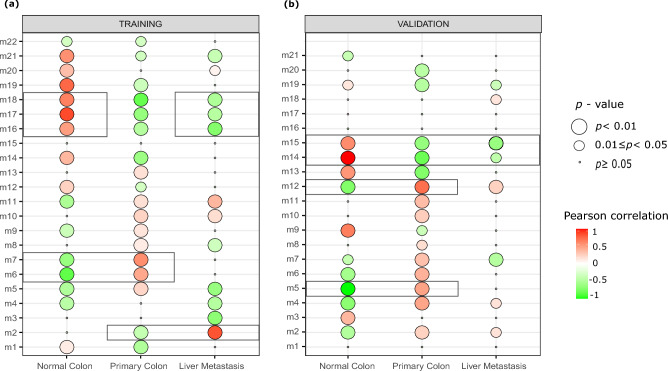
Identification of the most related co-expression modules (row) to specific phenotypes (column). The size of each circle indicates the *p*-value, while the color represents the Pearson correlation. (**a**) The correlation heatmap for the training dataset. (**b**) The correlation heatmap for the validation dataset. The original heatmaps of the WGCNA method are reorganized using the ggpubr R-library^19^.

Figure 1a indicates the correlation coefficient and p-value that represent the relationship between the respective module eigengenes (in rows) and sample phenotype (in columns) for the training set. A dynamic tree-cutting technique was utilized to identify modules that have similar gene expression profiles^18^. In this regard, a threshold of 0.25 height cut was set which corresponds to a correlation of 0.75. The modules with similar expression profiles were combined based on this threshold, resulting in 22 different modules. After examining the correlation and *p*-values, we selected six significant modules: the m2 module is for the liver metastasis from the primary CRC phenotype; m6 and m7 represent the primary CRC phenotype; m16, m17, and m18 are significant modules for the liver metastasis from the normal colon tissue phenotype.

The significant modules were merged to represent related genes into a main module for each phenotype. The members of these main modules show opposite mRNA expression patterns, e.g. the module members are down-regulated in normal colon samples, same genes are up-regulated in primary CRC samples. Supplementary Table S1 shows the number of genes in the main module of each phenotype. A vast number of genes (#7537) were observed in the “primary from normal” module, then followed by “metastasis from normal” (#2453) and “metastasis from primary” (#733) modules.

### Network clustering and submodule selection

The tissue-specific interaction networks of the selected three phenotypes were constructed based on related tissue. For the “metastasis from primary” module, its functional interaction network (FIN) was constructed on the liver tissue-specific network; for the “primary from normal” module, the FIN was constructed on the colon tissue-specific FIN; for the “metastasis from normal” module, the FIN was constructed on the liver tissue-specific FIN. The total number of genes and interactions in these FINs are given in Supplementary Table S2. Then network-based clustering algorithms run on these FINs. The performance of each clustering algorithm was calculated with evaluation metrics. The obtained results for the three phenotypes are summarized in the following sections.

#### Submodules for metastasis from primary colon samples

Markov clustering (MCL), fuzzy neighborhood (FN), spectral clustering algorithms as well as Infomap and Label Propagation (LP) were used in the FIN created for this phenotype. Five clustering algorithms run on the same FIN. The performance of each algorithm was evaluated using both internal and biological metrics, these evaluations are summarized in Supplementary Fig. S2. Considering evaluation metrics, the LP and Infomap algorithms achieved the best clustering results. The submodules detected by these two algorithms were re-evaluated with their individual Biological Homogeneity Index (BHI), Wang Biological Process (Wang-BP), and Molecular Function (Wang-MF) metrics. First, submodules with the highest BHI, Wang-BP, and Wang-MF values were analyzed, additionally, the presence of significantly regulated genes in the relevant submodule was considered. Accordingly, the analysis was applied to submodules given in Supplementary Table S3 for biomarker selection.

As a result of these analyses, the submodules 1, 12, and 13 of the Infomap algorithm and the submodule 2 of the LP algorithm were determined for further biomarker analysis. When all these submodules are considered, 57 genes represented increased expression values in metastatic samples compared to the primary CRC.

#### Submodules for primary from normal samples

All clustering algorithms were used in the FIN constructed for the phenotype of primary CRC developed from normal colon samples (Supplementary Fig. S3). FN and Spectral algorithms led to the best results. The submodules detected by these two algorithms were re-evaluated by metrics as given in Supplementary Table S4. As a result, submodules 2 and 7 in the FN algorithm and submodules 2 and 16 in the Spectral algorithm were chosen for further analysis. Within different submodules, 120 genes have increased expressions in primary CRC samples compared to the normal colon group. On the other hand, 9 genes showed decreased expressions in the same patient groups.

#### Submodules for metastasis from normal samples

Five clustering algorithms were applied to identify biomarker genes that play an important role in liver metastasis development from normal colon samples (Supplementary Fig. S4). We observed that LP and Infomap algorithms created too many clusters having few members. Since the results of the FN algorithm are quite consistent, the most significant submodules formed by this algorithm were selected for further analysis. For this process, the submodules detected by the FN algorithm were re-evaluated by biological metrics summarized in Supplementary Table S5; the presence of genes that changed significantly in the relevant submodule was also considered. As a result, submodules 1, 4, 5, and 12 were found to be significant. When these submodules were evaluated, 107 and 10 genes showed increased and decreased expressions, respectively in metastatic samples compared to the normal colon group. Supplementary Table S6 lists the genes in all key submodules identified by the clustering algorithms in the relevant phenotype.

### Validation set analysis

Statistically significant modules were also determined by applying WGCNA to the validation dataset. The Pearson correlation and *p*-values of the selected modules are given in Fig. 1b.

Four significant modules were extracted. Two of modules were in the "primary colon developed from normal colon tissue" phenotype (m5, m12), and two of them were in the "primary colon and metastasis developed from normal colon tissue" phenotype (m14, m15). Members of modules associated with the same phenotype were combined and two giant modules were obtained, the information on these modules is given in Supplementary Table S7.

Based on relevant phenotypes, the genes in the significant modules identified by WGCNA on the validation and training datasets were compared, and common genes within the same phenotypes in both datasets were extracted. Supplementary Table S8 summarizes the number of these common genes. Supplementary Table S9 lists their names, gene expression levels, and related phenotypes.

### Biomarker selection

A gene enrichment analysis was carried out for common genes given in Supplementary Table S9. Final biomarkers were chosen from genes with increased or decreased mRNA expression profiles and involved in significant biological processes or pathways.

#### Biomarkers for primary CRC

Significantly expressed genes for the development of primary CRC from normal colon tissue were explored. Increased levels of mRNA expression were observed for all genes. Based on the gene set enrichment analysis, significant KEGG pathways include *the cell cycle, p53 signaling pathway*, and *cellular senescence*. Important biological processes cover *positive regulation of the cell cycle process*, *regulation of G2/M transition of the mitotic cell cycle*, *G1/S transition of the mitotic cell cycle*, *regulation of signal transduction by p53 class mediator*, *regulation of cell population proliferation*, and *regulation of the apoptotic process*. Details regarding the gene set enrichment analysis for primary CRC are provided in Supplementary Table S10.

#### Biomarkers for primary CRC and metastasis development

Genes that are significantly expressed in the development of primary CRC and metastasis from normal colon tissue were examined. Based on the gene set enrichment analysis, important biological processes include *negative regulation of ERK1 and ERK2 cascade* and *regulation of cell migration*. Important hallmarks of cancer terms include *KRAS signaling up* and *IL-6/JAK/STAT3 signaling*. DUSP10, CLDN1, SERPINA3, and SLPI genes, which are involved in important biological processes, show increased expression in both datasets. It was determined that SLC9A3R1, CEACAM1, IL10RB, and IL1R2 genes showed decreased expression in both training and validation datasets for primary CRC and metastasis. Details regarding the gene set enrichment analysis for primary CRC and metastasis are provided in Supplementary Table S11.

### Therapeutic drugs

The biomarkers summarized Supplementary Table S12 were mutually observed in significant modules for both training and validation datasets, thus they were provided as input to search for drugs that can therapeutically target these proteins. The drugs, that can therapeutically target the given biomarkers, were searched from the Drug-Gene Interaction Database (DGIdb)^20^ as explained in the method section.

#### Drugs suggested for treating primary CRC

**Figure 2:**
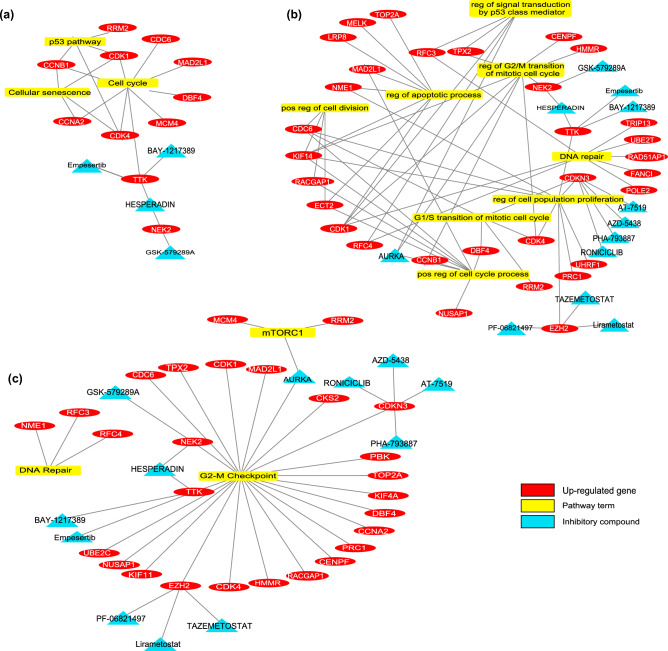
The pathway-gene-drug relations were obtained after gene enrichment and drug screening for normal versus primary colon cancer groups. (**a**) KEGG pathway, (**b**) GO-Biological Process, and (**c**) Cancer Hallmark term.

There are 61 biomarker genes with increased expressions in the normal colon and primary CRC samples. As a result of drug screening, many drugs were retrieved to target these proteins. Figure 2 shows the enrichment results of target biomarkers, associated pathways / processes and their therapeutic drugs. The large drug list was re-scanned in the literature by using "tumor growth”, “proliferation”, “cell death”, “apoptosis”, “autophagy”, “G1-S arrest”, “invasion”, “EMT mechanism”, or “colorectal cancer” keywords. As a result of all these analyses, more specific protein targets and drugs were identified. Supplementary Table S12 shows the limited drug list. Among these drugs, the BAY-1217389 and Hesperadin compounds, which inhibit TTK protein kinase, were considered for in-vitro experiments to show their efficacy in the primary CRC cell lines.

#### Drugs suggested for treating liver metastatic CRC

**Figure 3:**
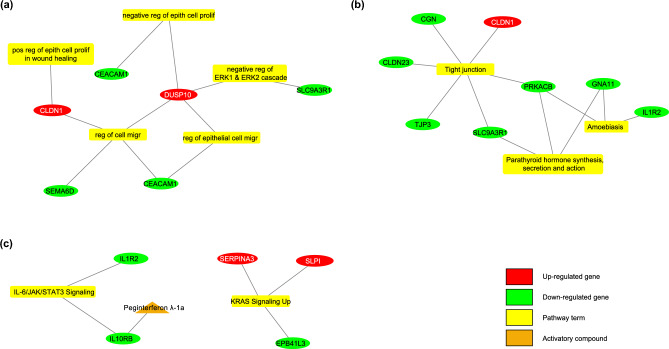
The pathway-gene-drug relations were obtained after gene enrichment and drug screening for normal versus primary colon cancer versus liver metastasis groups. (**a**) GO-Biological Process, (**b**) KEGG pathway, and (**c**) Cancer Hallmark term.

In the sample group of normal colon, primary CRC, and liver metastasis, there were 42 genes (34 of them with decreased, 8 of them with increased mRNA expression). Figure 3 shows the enrichment results of biomarkers and their therapeutic drugs. The drugs targeting biomarkers, which work in cancerization and metastasis processes, have been investigated. There was no inhibitory group that targets proteins with increased expression levels. Among the biomarkers with decreased expressions, the “Peginterferon λ-1a” from the activator group was detected for targeting IL10RB, which is a member of the IL-6/JAK/STAT3 signaling pathway (Supplementary Table S13). This agent was used to prove its in-vitro efficacy in the liver metastatic CRC cell line.

### Experimental validation

#### Hesperadin decreases cell viability in short-term cytotoxic effect

**Figure 4:**
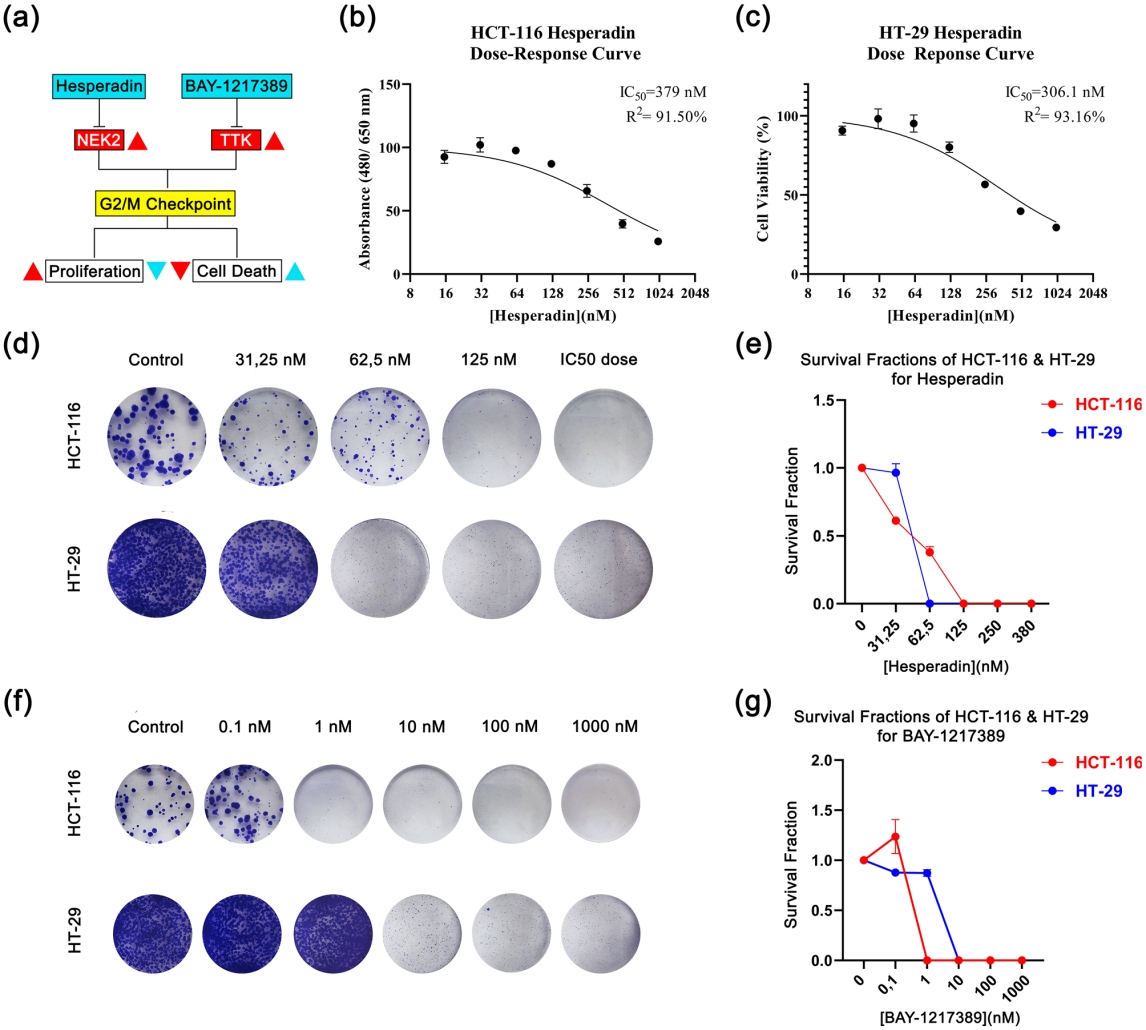
Cell viability and survival graphs in response to drug treatments. (**a**) A basic graphical abstract of in the vitro pipeline indicating hesperadin and BAY-1217389’s mechanism of anti-proliferative and cytotoxic effects is shown. While red color represents cancer status, light blue serves as treatment status. The direction of the arrows points out an increase (▲) and a decrease (▼). (**b)** Hesperidin dose–response curve in HCT-116. (**c**) Hesperadin dose response curves in HT-29. Colony images along with relative dose/survival fraction graphs are shown for HCT-116 (**d**,**e**) and HT-29 (**f**,**g**).

In-silico analysis predicts that TTK and NEK2 are promising targets to treat the proliferative behavior of CRC. The network analysis indicates these proteins are related to cell division and proliferation. Therefore, the in-silico model suggests that their potent inhibitors Hesperadin and BAY-1217389 (BAY-12) could decline cancer progression (Fig. 4a).

First, we investigated if Hesperadin and BAY-12 could decrease cell viability of grade II-III human colon carcinoma lines, HT-29 and HCT-116. The cells were treated with 0.1–1000 nM concentrations of these agents and cell viability was measured by formazone forming WST-1 assay. The result points out that Hesperadin has a potent effect on the viability. Its IC_50_ values are 379 nM and 306.1 nM for HCT-116 and HT-29 respectively (Fig. 4b,c). Whereas Hesperadin abates cell viability of both lines within this dosage range in 48 h, BAY-12 indicates no cytotoxic effect. However, we hypothesized that BAY-12 could have a cytostatic effect in the long term due to its possible relation with the cell cycle.

#### BAY-1217389 led to a decline in colony formation in 14-days (long-term period)

We performed a colony formation assay to estimate if there is a long term cytostatic effect on the cell reproductive system. Even though BAY-12 did not cause a cytotoxic effect in 48 h in the WST-1 viability assay, it decreased the colony forming capacity. In response to 10 nM of this agent, there was no colony counted after 14 days. Similarly, Hesperadin inhibited clonogenic behavior in a dose-dependent manner. HCT-116 cells tolerated this agent better than HT-29, consistent with their IC_50_ values. The survival fraction declined progressively at 15 to 62.5 nM concentration and the colonies cleared away at 125 nM for HCT-116 (Fig. 4d,e). There were no colonies observed in response to a 62.5 nM dose of hesperidin for HT-29 (Fig. 4d,e). Thus, we suggest while HT-29 is more vulnerable to Hesperadin treatment, BAY-12 affects both cells at lower doses (10 nM) (Fig. 4f,g).

Even though both Hesperadin and BAY-12 have cytotoxic or cytostatic effects on cell viability in CRC, BAY-12 was more likely cytostatic in long-term periods considering low response to WST-1 while high performance in colony formation. Both candidates have anti-proliferative effects on CRC.

#### Hesperadin and BAY-1217328 have anti-proliferative effect

**Figure 5:**
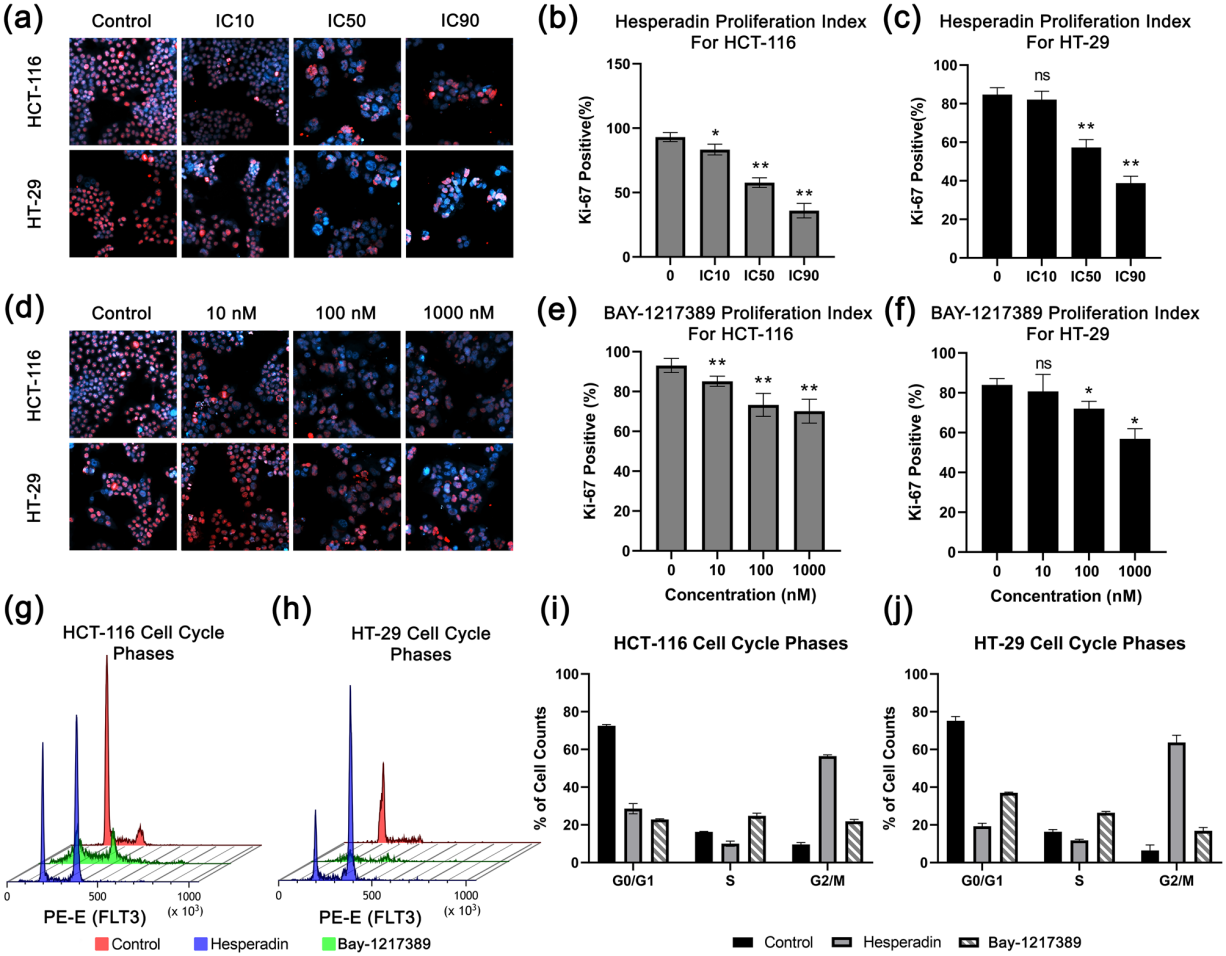
Proliferation and cell cycle graphs. Immunofluoresense images of Ki-67 (red) as proliferation marker and Hoescht-33342 as counter staining (blue) in response to (**a**) hesperadin and (**d**) BAY-1217328 treatments are presented. Bar graphs represent proliferation indexes for each doses according to Ki-67 positive cell count percentage in hesperadin treated for (**b**) HCT-116, for (**c**) HT-29 and BAY-1217389 treated groups for (**e**) HCT-116, for (**f**) HT-29. Cell cycle histograms are stand for (**g**) HCT-116 and (**h**) HT-29. Red indicates control, blue is hesperadin treated and green is BAY-1217389 treated cells. Bar graphs represent cell cycle phases percentages of cell counts are shown for (**i**) hesperadin and (**j**) BAY-1217328 treatments. “n.s” stands for non-significant. Asterisk (*) indicates *p*-value < 0.05, and double asterisk (**) means *p*-value < 0.01.

The effect on viability was researched whether it was an anti-proliferative or cytotoxic response. Immunofluorescence Ki-67 stainings were performed to evaluate proliferative cells. The proliferation index of both lines was decreased in a dose dependent response against Hesperadin. While the Ki-67 positive cells were 80% for control samples, it was 57.25% and 52.68% for IC_50_ concentrations of this agent in HT-29 and HCT-116 cells, respectively (Fig. 5a–c). Although Hesperadin shows a slightly more potent cytotoxic effect on HT-29 in account of WST-1 and colony formation assays, there is no statistical difference in Ki-67 count.

Whereas there were no IC_50_ values measured through WST-1 viability assay in 48 h for BAY-12, 10 nM, or higher doses abated colony formation. Henceforward, the anti-proliferative effect of this agent was investigated in a range of 10–1000 nM doses. The proliferation indexes of control samples were 83.91% and 93.06%. This ratio was reduced to 72.03% and 73.30% in response to 100 nM of BAY-12 treated samples for HT-29 and HCT-116 cells, respectively (Fig. 5d–f). At higher doses, HT-29 was more sensitive to BAY-12. 1000 nM maximum dose decreased Ki-67 positive cell to 56.90% for HT-29 while this highest concentration diminishes the signal no more than 70.13% for HCT-116.

#### Hesperadin and BAY-1217389 arrest cell cycle at the G2/M phase

Because the Ki-67 biomarker indicates that Hesperadin and BAY-12 have a degree of anti-proliferative effect on cell viability and the identified therapeutics target cell cycle regulators TTK and NEK2, we investigate in which phase they hinder the cell cycle. To characterize the anti-proliferative mechanism of these drugs, the DNA amount of pre-treated cells stained by Rnase I/ Propidium Iodide (PI) assay after 48 h incubation and measured in flow cytometry. As shown in Fig. 5g–j, these candidates arrest cell cycles at the G2/M phase in HCT-116 and HT-29 cell lines. Hesperadin has a more potent effect on this behavior (56.9%, 61.0% for HCT-116 and HT-29, respectively) than Bay-12 (22.6%, 18.1% for HCT-116 and HT-29, respectively).

#### IL-29A inhibits the metastatic behavior in colorectal carcinoma

In-silico analysis also predicts the agent Peginterferonλ-1A for metastatic CRC. Network analysis suggested that the decrease in the expression level of IL10RB in the IL-6/JAK/STAT3 pathway in metastatic CRC patients may be associated with the metastatic phenotype. We thus verified the data derived from in-silico analysis under in-vitro conditions.

**Figure 6:**
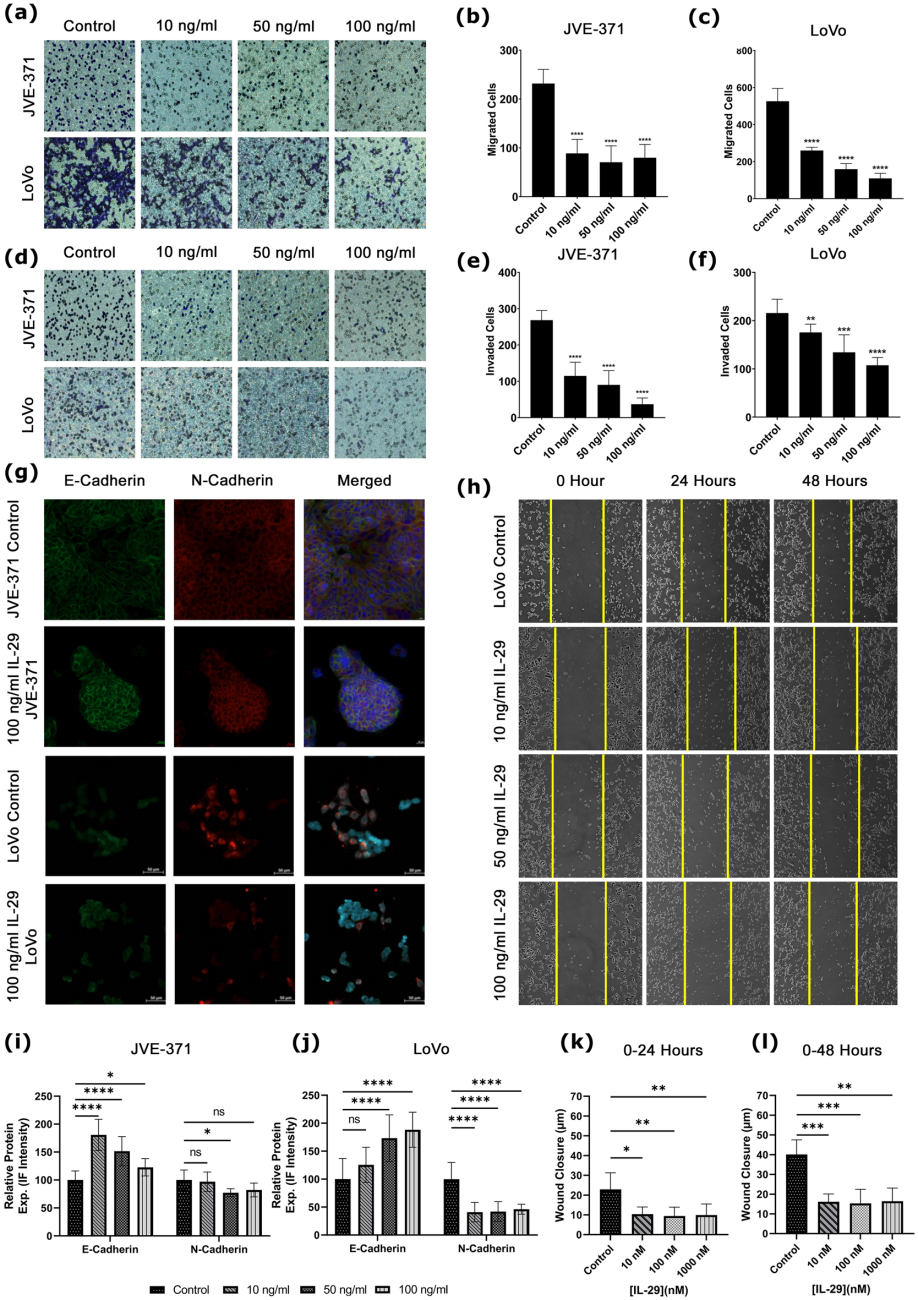
Migration, invasion and EMT capacity in response to IL-29. (**a**) Cells migrated and (**d**) invaded the lower membrane in transwell assay treated with IL-29 doses and control groups stained with crystal violet (dark blue). Bar graphs represent migrated cells by doses of IL-29 for (**b**) JVE-371 and (**c**) LoVo. Similarly, invaded cells are presented for (**e**) JVE-371 and (**f**) LoVo. (**g**) IL-29 metastatic cell lines are stained with EMT markers. Green stands for E-Cadherin, Red is N-Cadherin, and Blue is Hoescht 33,342 (as counterstain). Relative protein expression is analyzed according to IF intensity and shown in bar graphs for (**i**) JVE-371 and (**j**) LoVo. (**h**) Wound healing images are presented. Wound closure in time (**k**) 0–24 h and (**l**) 0–48 h are shown. Also, Bar graphs represent cell cycle phases percentages of cell counts are shown for hesperadin (**i**) and BAY-1217389 (**j**) treatments. “n.s” stands for non-significant. Asterisk (*) indicates p < 0.05, double asterisks (**) means *p*-value < 0.01, Triple asterisks (***) means *p*-value < 0.001, and quadruple asteriks (****) indicates *p*-value < 0.0001.

To validate the effect of the agent Peginterferonλ-1A for metastatic CRC, we investigated its non-pegylated form, interferonλ-1A (IL-29A). Firstly, the efficiency of IL-29A on migration and invasion capacities of LoVo and JVE-371 metastatic CRC cell lines was identified by transwell assay. IL-29A was treated with 10, 50, and 100 ng/ml concentrations on LoVo and JVE-371 cell lines at 48 h. In the lymph node metastatic CRC LoVo cell line, IL-29A decreased the migration rate at 10, 50 and 100 ng/ml concentrations, respectively, compared to the TGFβ-induced control group (Fig. 6a–c, p-value < 0.0001). IL-29A also decreased invasion rate compared to the TGFβ-induced control group in increasing concentrations on the LoVo cells (Fig. 6d,f, p-value = 0.0065, *p*-value = 0.0003 and *p*-value < 0.0001). In the liver metastatic colorectal carcinoma JVE-371 cell line, migration, and invasion rates were decreased compared with the TGFβ-induced control group (Fig. 6d,e p-value < 0.0001).

Afterwards, epithelial-mesenchymal transition (EMT) was evaluated, since EMT is a highly dynamic process that allows cells to transition from the epithelial form to the mesenchymal form. Therefore, EMT leads to the initiation of the metastatic cascade^21^. TGF-β induced JVE-371 cells to induce EMT showed a significant decrease in the amount of E-cadherin (Fig. 6g, p-value < 0.0001) and a significant increase in the amount of N-cadherin (Fig. 6g,i p-value < 0.0001) compared with the non-TGF-β induced group. A significant increase in the amount of E-cadherin was observed when IL29A was applied to TGF-β-induced JVE-371 cells (Fig. 6g,i p-value < 0.0001, *p*-value = 0.0041). At the same time, a significant decrease was observed in the amount of N-cadherin at 50 and 100 ng/ml concentrations (Fig. 6g,i, p-value < 0.0003, *p*-value < 0.0274), without any change at 10 ng/ml concentration (Fig. 6i. *p*-value = 0.5951). According to the immunofluorescence staining results, no statistically significant change was observed in LoVo cells at 10 ng/ml IL29A concentration compared to the TGFβ-induced control group. However, a statistically significant increase in the amount of E-cadherin was observed at 50 ng/ml and 100 ng/ml concentrations (Fig. 6g,j, P = 0.4706, *p*-value = 0.0012, and *p*-value < 0.0001). Likewise, a significant decrease was observed in the amount of N-cadherin, an indicator of mesenchymal phenotype, at increasing concentrations of IL29A in LoVo cells compared to the TGFβ-induced control group (Fig. 6g,j, *p*-value < 0.0001).

To investigate migrative phenotype, a wound healing assay was established in addition to a transwell migration assay. We monitored wound closure distance (µm) of LoVo cells for 48 h in control and IL-29 treatment groups. IL-29 treatment decreased the capacity of wound closure of LoVo cells at 24 and 48 h, indicating that this treatment decreased migrative potential (Fig. 6h,k,l). The data is correlated with the results of the transwell migration assay. JVE-371’s growth pattern is not suitable for wound healing assay, therefore we couldn’t monitor it in this experiment.

Overall, we investigated the metastatic phenotype by transwell migration invasion capacity, wound closure potential, and mesenchymal phenotype. The results indicate that in silico predicted IL-29A attenuates metastatic phenotypes in lymph metastatic LoVo and liver metastatic JVE-371 cells.

## Discussion

In this study, we present a general pipeline that defines and optimizes new therapeutic targets for CRC treatment through in-silico workflow and experimental research to evaluate their therapeutic potential in-vitro. We conducted bioinformatic analyses to identify potential anti-proliferative and anti-metastatic agents for the treatment of CRC. Based on our analysis, we selected three agents: Hesperadin and BAY-1217389 as anti-proliferative agents, and IL-29A as an anti-metastatic therapeutic.

Of the three agents Hesperadin, an inhibitor of Aurora kinases, has been studied in various cancers including ovarian, kidney, pancreas cancers, and leukemia. It can induce cell cycle arrest and impair spindle assembly checkpoint in cancer cells, leading to reduced cell proliferation and even increased sensitivity to radiation therapy^22–25^. To the best of our knowledge, this study is the first one reporting the anti-cancer effect of Hesperadin on CRC cells. Our validation pointed out that Hesperadin has both cytotoxic and anti-proliferative effects on HCT-116 and HT-29 cell lines. Together with Hesperadin as an inhibitor of Aurora kinases, our model also suggested that it could potentially suppress NEK2 and TTK, which (Mps1) are mitotic regulators in the G2/M phase of cell division. Since hesperadin and BAY-1217389 target these two regulators of the G2/M checkpoint that are associated with colon cancers according to our research panels, we proceeded to examine the in-vitro effects of Hesperadin on cell division and proliferation. We observed that Hesperadin arrests the cell cycle at G2/M phase. Various studies in other cancer types support our results^24,25^. Hesperadin has been reported to have a cytotoxic effect on cells by causing G2/M phase retention and apoptosis in pancreatic cancer and uveal melanoma^24,25^. Similarly, we suggest that hesperadin causes an anti-proliferative effect and cytotoxicity through G2/M arrest and impairment of spindle assembly in CRC.

NEK2 is an intriguing mitotic regulator that offers promising therapeutic potential. Its overexpression has been strongly linked to histological differentiation, advanced TNM stage, lymph node metastasis, and tumor invasion^26,27^. Notably, Lu et al. have highlighted that elevated levels of NEK2 protein in CRC indicate the presence of malignant behavior^28^. Recent knockdown-based studies suggested that NEK2 inhibition could serve as a novel combination treatment^29,30^. While certain inhibitors like MBM-5 have demonstrated the ability to induce apoptosis and polypleoid nuclei through NEK2 inhibition, none of these inhibitors have progressed to clinical trials for CRC^31^. The majority of irradiation and chemotherapy induced polyploid giant cancer cells (PGCC) goes into cell death or lose proliferative capacity. However, the PGCC population is also related to relapse and therapy-resistance^32^. Recently, NEK2 inhibition has been found to impair oncogenesis and radioresistance in cervical cancer^33^. Therefore, combination approaches of radio- and chemotherapy with hesperadin would be promising to decrease relapse and resistance. Consequently, the pursuit of new drug candidates targeting NEK2 continues unabated.

Recent studies suggest that inhibiting monopolar spindle 1 (MPS1) may be a promising new approach to treating cancer. The expression of the TTK gene is significantly reduced or absent in quiescent cells and tissues with a low proliferation index^34^. In contrast to conventional antimitotic therapies, emerging evidence suggests that the strategy is not to arrest cell proliferation, but rather to inactivate the spindle assembly checkpoint. This inhibition therapy leads to aneuploidy and ultimately cell death^35,36^. Our in-silico model suggests MPS1 as a promising target in CRC. Our experimental validation indicates that BAY-1217389, an MPS1 inhibitor, has a long-term effect viability of HCT-116 and HT-29 cell lines supporting the idea. Yet, there are a limited number of studies indicating that inhibiting TTK leads to the promotion of aneuploidy and induction of cell death in CRC^37^. Schulze et al. reported that BAY-1217389 also inhibits HT-29 cell viability at IC_50_ 62 nM in 48 h^36^. Also, ovarian tumor xenograft models showed moderate efficacy as a monotherapy in preclinical studies^38^ and good tolerability without increasing toxicity when combined with paclitaxel in preclinical studies. However, a phase I clinical trial on the combination of BAY-1217389 with paclitaxel has shown considerable toxicity without a clear therapeutic window in breast and lung cancers^39^. Together with results in colon carcinoma, these studies suggest that further investigation is needed to optimize this promising approach and identify clinically viable combinations with BAY-1217389 for translation into the clinic.

The metastatic effect of INF-λ has not yet been fully clarified and there are opposite ideas about the effect of INF-λ in literature. Lee et al. claimed that IL-28A, also known as INF-λ2, induced cell migration by NF-κB-dependent matrix metalloproteinases-9 expression in bladder cancer^40^. Likewise, in different studies, it has been shown that INF-λ2 induces cancer cell migration and angiogenesis in mouse and canine carcinoma cells^41,42^. On the other hand, Gao et al. have shown that INF- λ1 (IL-29) suppressed invasion and increased autophagy in human osteosarcoma cells^43^. Similarly, Hubert et al. suggest that both the receptor and the IFN-λ correlated with good prognosis in breast cancer patients. Moreover, they point out that a conventional dendritic cell 1, which synthesizes INF- λ1, is negatively correlated with EMT transition^44^. To the best of our knowledge, we reported for the first time the effects of IL-29 on the metastatic phenotype of CRC. Our experiments revealed that IL-29 suppresses both migratory and invasive behavior for human lymph nodes and liver metastatic CRC cell lines. In addition, TGF-β induced epithelial-mesenchymal transition, which is an inducer marker of the metastatic process, is inhibited by IL-29. Recently, Zhang et al. claimed that type III interferons including IL-29 activate JAK1 and STAT1 signaling pathway to repress migration and invasion in breast carcinoma cell line MCF-7^45^. Similarly, our network analysis indicates that metastatic behavior is correlated to diminished function of the IL-6/JAK/STAT pathway due to decreased expression of IL10RB. Collectively, PEG-IFN-λ1a, which is the pegylated form of IL-29, would be promising anti-metastatic therapeutic to restore the protective function of IL-6/JAK/STAT pathway due to IL10RB activation in CRC.

This study proposes new targets for treatment of primary and liver metastatic CRC. The validations of BAY-1217389 and hesperadin revealed their anti-proliferative effects on HCT-116 and HT-29 cell lines. Additionally, they have the potential to arrest cell cycles at the G2/M phase. On the other hand, IL29A decreased the migration and invasion capacities of LoVo and JVE-371 metastatic CRC cell lines. Although these experiments have potential as alternative CRC treatment, there is a need for further investigation for optimization of the treatment protocol.

## Methods

### Data analysis

Tissue samples for normal colon, various stages of CRC, and liver metastases from CRC were retrieved from the GEO database^12^. Twelve datasets using the Affymetrix hgu133plus2 chip were selected. The first nine microarrays were used to train the model, and the other three were used for in-silico validation of the identified biomarkers. Table 1 shows the total number of samples for both the training and validation datasets. If it is provided in the GEO repository, the demographic data (sex, age) and sample type (normal, primary, metastatic, etc.) are summarized in Supplementary Table S14.

**Table 1:** Dataset details.

GEO ID	Normal colon	Primary colon	Metastatic liver	Training set	Validation set
GSE4107	10	12		✓	
GSE4183	8	15		✓	
GSE8671	32			✓	
GSE9348	12	12		✓	
GSE10714	3	7		✓	
GSE10961			18	✓	
GSE13471	4	4		✓	
GSE15960	6	6		✓	
GSE18105	17	28		✓	
GSE18462	2	2	2		✓
GSE37364	38	27			✓
GSE40367		8	7		✓
Training	92	84	18		
Validation	40	37	9		
Total	132	121	27		

Various preprocessing steps have been applied on the microarray samples. Firstly, the "rma" (Robust Multi-Array Average) method of the "affy" library in Bioconductor was used for the normalization of probes^46^. After this normalization process, the remaining probes were labeled by the gene "Entrez Identifier" and the probes with "NA" gene identifiers were eliminated. A gene can be represented by more than one probe in microarray chips. To resolve this issue, the median value of mRNA measurements was calculated for repetitive probes and this median value was assigned as the mRNA expression value for the relevant gene. Since each of the normalized data sets is produced from experiments performed by different laboratories, it is necessary to reduce their batch effect to obtain statistically more meaningful results. For this purpose, the "Combat" function in the "sva" package in Bioconductor is used^47^. Supplementary Fig. S5 presents data distribution before and after the removal of the batch effect. As the batch effect was reduced, it was observed that phenotype clusters showed better separation.

All these data preprocessing methods were also applied to the validation datasets by following the same steps. As a result, a total of 20,174 probes representing individual gene regions remained for 194 samples in the training dataset and 86 samples in the validation set.

### Construction of tissue-specific functional interaction networks

An integrated FIN was constructed using interaction data from the study of Linghu et al.^48^. This network uses protein pairs that participate in the same biological process. In this network structure, each node is a human protein, and an interaction (link) connecting two proteins shows their functional similarity. The FIN consists of 20,790 proteins and 21,952,150 interactions. The weight value of each interaction represents the similarity of biological function between two proteins, and these values range from 0 to 1. Proteins with very low functional similarity (those within the 0–0.1 range) were excluded. After this filtering, 15,002 proteins and 334,225 interactions remained in the FIN.

Proteins that are not synthesized in the relevant healthy (colon or liver) tissue and the connections between them were extracted from the original FIN by using the "Tissue Atlas" data from the "Human Protein Atlas" Project^49^. Thus, noise that may be caused by irrelevant proteins was prevented; liver and colon-specific FINs were obtained. As a result of this analysis, the liver-specific FIN covered 13,184 genes and 200,492 common interactions. The colon-specific FIN contained 14,486 genes and 234,189 interactions. Then, submodules on these tissue-specific networks were determined by using the "components" function in the "igraph" library^50^. For liver tissue, 27 submodules were identified, with the largest module containing 10,359 genes and 200,458 links. For colon tissue, 24 submodules were identified, with the largest module containing 11,355 genes and 234,152 links. The following analyses were continued with the largest modules obtained for both tissues.

### Weighted gene co-expression network analysis

The WGCNA method was used to construct a gene co-expression network for 194 samples in the training set and 86 samples in the validation datasets, individually. It was aimed to determine the gene expression modules showing the highest correlation with the considered phenotypes of patients.

First, the "pickSoftThreshold" function was used to select an appropriate soft-thresholding power for network construction by calculating the scale-free topology fit index for several powers^17^. Next, a weighted adjacency matrix representing a gene co-expression network was constructed using this threshold value. Additionally, the adjacency matrix was converted into a topological overlap matrix (TOM) to estimate gene connectivity in the network. The hierarchical clustering of the TOM-based dissimilarity matrix calculated by subtracting the TOM from 1 (with the "hclust" function) was used. The clustering tree was pruned with the dynamic tree-cutting method from the “dynamicTreeCut” package, and significant modules with similar gene expression profiles were identified and labeled by a different color^18^. The original heatmap of the WGCNA method, which shows the relationship between the respective module eigengenes and sample phenotypes, is reorganized using the ggballoonplot function of the ggpubr R-library^19^.

### Implementation of clustering algorithms

Genes in each selected module were pooled and directly mapped on colon or liver specific FINs. For this process, the phenotypes associated with each module were considered. The "ego" function in the "igraph" library is used for this filtering process^50^. A network structure in which the genes in each module are directly adjacent/neighbor to each other in the FIN was considered for further analysis.

When analyzing complex and large networks, traditional clustering methods that only consider genes can lead to insufficient module generations. For this reason, network-based clustering methodologies, which both consider the genes and interaction weights in the network, were used to analyze the functional properties of the networks and identify the interacting genes in the modules. There are many algorithms for network clustering, as well as several different libraries available for their implementation. Since colon and liver specific FINs comprised more than 10,000 nodes and more than 200,000 weighted edges, this required us to choose the algorithms we used based on their runtime, ability to handle large data sets and return a reasonable number of clusters. MCL, FN, and spectral clustering algorithms were run separately on the same network (colon specific FIN or liver specific FIN) to find the most significant submodules in terms of biological functions. Due to a lack of finding statistically significant results for phenotypes of “metastasis development from normal colon tissue” and “metastasis development from primary colon cancer”, Infomap and LP clustering algorithms were also applied for these phenotypes.

The MCL algorithm runs using libraries in Python and R languages. Since the highest modularity score was obtained when the inflation operator was "1.2" and the expansion operator was "2", the algorithm runs with these values. For the fuzzy neighborhood algorithm, the "cluster" function in the “ProNet” package was used by setting the method parameter as "FN". For the spectral clustering algorithm, the "SpectralClustering" function in the Python "Sklearn" library was used. The algorithm runs with the following parameter values: affinity as "precomputed", assign_labels as "discretize", and random_state as "0". The "cluster_infomap" function in the "igraph" library was used for the infomap algorithm, and the "cluster_label_prop" function was used for the LP algorithm.

The performance of each clustering algorithm was evaluated using both internal and biological metrics. Internal evaluation metrics used are modularity and silhouette. Biological metrics assess the capability of a clustering algorithm to produce more biologically significant gene modules. The biological metrics are the BHI, Wang-BP, and Wang-MF Index. Details about the metrics used were described in our previous study^51^. The submodules that provide the optimum clustering results were re-evaluated with the individual BHI, Wang-BP, and Wang-MF criteria, and finally the submodules with the highest biological evaluation criteria were selected for further analysis.

### Gene expression analysis

Gene expression analysis was also performed to show the mRNA changes between patient groups (e.g. normal colon vs. primary colon) based on the phenotypes of the modules obtained by WGCNA. In this analysis, both the student’s *t* test and fold change calculation were applied. The *p-*values were corrected by the “False positive rate (FDR)” method. Statistically significant gene lists were obtained by filtering genes with absolute fold change values > 1.0 and FDR < 0.05. Next, the mutual genes were identified by intersecting genes in the selected modules and statistically significant ones for the same phenotype group. Then, significantly expressed genes within these selected submodules were proposed for candidate biomarkers.

### In-silico validation of biomarkers

The same systems biology methods were applied on the validation dataset and the potential of biomarker genes identified on the training set was re-evaluated. After the data preprocessing stage, WGCNA was performed with the expression data of 20,174 genes for a total of 86 patients in the validation set. The genes in the significant modules obtained from the WGCNA applied on the validation samples and the sub-modules obtained from the analysis of the training dataset were compared based on the relevant phenotypes, and the mutual genes observed in the same phenotypes in both datasets were determined. The EnrichR enrichment library identified the significant biological processes (GO-BP, GO-MF) and signaling pathways (KEGG) of the mutual genes by setting the FDR < 0.05 threshold. To determine expression changes of genes obtained in the modules of WGCNA, the genes showing statistically significant expression changes were selected. Finally, the biomarkers were identified by selecting genes with significant mRNA expressions enriched within the related biological processes and pathways.

### Therapeutic drug identification

DGIdb combines information from 41 different databases and presents drug and target protein interactions both as a web interface and as an R package^20^. Although drug interactions are defined in many modes of actions, we grouped them into two main groups. The activatory mode covers drugs that increase the biological activity or expression of a target. The inhibitory mode includes drugs that reduce the biological activity or expression of a target. The inhibitor group contains antagonist, antibody, antisense oligonucleotide, blocker, cleavage, inhibitor, inhibitory allosteric modulator, inverse agonist, negative modulator, partial antagonist, and suppressor expressions; while the activator contains agonist, chaperone, cofactor, inducer, partial agonist, positive modulator. The drugs in the activator group were used for targets with decreased expression, and the inhibitor group worked for targets with increased expression.

As a general search approach, targeting oncogenes with increased expression in tumor tissues is a more strategic method, as it is known to produce fewer side effects than targeting tumor suppressor genes with decreased expression in cancer. Therefore, compound screening was first focused on genes with increased expression. If a candidate drug was not found in this screening, the target genes with decreased expression were searched within the activator group drugs. The entire search process was done using the rDGIdb R package of the DGIdb.

### Cell culture

HCT-116 (CCL-247, ATCC), HT-29 (HTB-38, ATCC), Lovo (CCL-229, ATCC), and JVE-371 (ACC 825, DSMZ) cell lines are cultured to model in vitro colon adenocarcinoma and its liver metastasis. HCT-116, HT-29, and LoVo lines are cultured in complete media consisting of DMEM/Ham’s F12 media (E0500-210, Cegrogen) complemented with 10% fetal bovine serum (FBS) and peniciline/streptomycin. Metastatic JVE-371 cells were grown in RPMI-1640 (E0500-360, ATCC) enriched with 20% FBS. All lines were cultured at 37 °C with a humidified 5% CO_2_ atmosphere.

### Cell viability assay

Cell viability was measured with formazone forming WST-1 assay according to the manufacturer's protocol. Cultured cells were trypsinized and counted with trypan blue in hemocytometer and seeded 104 cells per well. After 24 h of incubation to let the cells attach and get their morphology, cells are treated with 0.1–1000 nM concentrations of drug candidates for 24 and 48 h to estimate IC50 values. Absorbance values were measured at 430 nm with 650 nM reference wavelength.

### Colony formation assay

To estimate cytotoxicity and cell reproductive death induced by candidate agents, a colony formation assay was established. 103 Cells were seeded per well in 6-well plates and cultured in complete media supplemented with 5 doses in the range of minimum to IC50 doses for 14 days. Every third day culture media were renewed. Cells were fixed with ice-cold methanol and stained with crystal violet (0.5% v/v). Colonies were photographed and counted with the ImageJ software.

### Immunofluorescence microscopy (IF)

Immunofluorescence imaging was established to display and measure proliferative and mesenchymal phenotypes in response to drug candidates. Cells were seeded on 96-well plate, washed three times with PBS, and fixed with ice-cold methanol. Then they were blocked with 1% Bovine Serum Albumin (BSA), followed by incubation with primer antibodies (rabbit pAb anti-67, ABCAM; rabbit mAb N-Cadherin, Thermofisher Sc.; Mouse mAB E-Cadherin, Thermofisher Sc.) overnight. Cells were washed three more times and treated with anti-rabbit or anti-mouse goat seconder antibodies (anti-rabbit goat Alexa Fluor 568, Thermofisher Sc.; anti-mouse goat Alexa Fluor 488, Thermofisher Sc.; dilution 1:1000) for 1 h at room temperature.

### Cell cycle assay

Cell cycle phases were measured by RNase I/ Propidium Iodide (PI) assay in response to drug candidates. Following the drug treatment by hesperadin and Bay-1217389, cells were harvested and fixed with drop-based ice-cold 70% ethanol while vortexing the samples. Fixed samples were washed three times with cold PBS to remove excessive ethanol before flow cytometry analysis. Samples were treated with RNase A (0.5 µg/ml)/PI and incubated overnight. Samples were analyzed by flow cytometry.

### Wound healing

Cells were seeded in 24-well plates and cultured until they covered the well surface to nearly 80% confluency. Then they were stretched by 100 µl pipette tips to produce wounds. Culture media refreshed with supplemented complete media with IC10 and IC50 drug doses. The closure was observed after 0-, 24-, and 48-h incubation with an inverted microscope (Zeiss Axio Vert.1, Germany). The images of each well were captured three different points and repeated independently three times.

### Transwell migration and invasion assay

Transwell assays were performed in 24-well inserts with 8.0 µM pore size (a) for cell migration and invasion. Before the experiment was established, cells were starved in serum-free media overnight. In addition, upper wells were coated with 200 µl of matrigel to model invasion through the extracellular matrix. 5 × 104 cells were seeded in upperwells of inserts in serum-free media (three replicates for each dose). Then, Inserts were placed into complete media (with 10% FBS). Both migration and invasion experiments were observed for 24 and 48 h. Cells in the upper chambers were removed and migrated/invaded cells were fixed with ice-cold methanol and stained with crystal violet (0.5% v/v). The cells were imaged and counted with an inverted microscope.

### Supplementary Information

Supplementary Information.

Supplementary Table S6.

Supplementary Table S9.

Supplementary Table S10.

Supplementary Table S11.

Supplementary Table S14.

## Data Availability

All patient samples are available from the GEO database (accession numbers: GSE4107, GSE4183, GSE8671, GSE9348, GSE10714, GSE10961, GSE13471, GSE15960, GSE18105, GSE18462, GSE37364, GSE40367).
