# Supplementary material for: New treatment alternatives for primary and metastatic colorectal cancer by an integrated transcriptome and network analyses
Source: Sci Rep. 2024 Apr 16;14:8762. doi: 10.1038/s41598-024-59101-8 (PMC11021540; doi:10.1038/s41598-024-59101-8)
Supplement: Supplementary file 1 — Supplementary Information. [file 41598_2024_59101_MOESM1_ESM.pdf]

Supplementary Information

New treatment alternatives for primary and metastatic colorectal cancer by an integrated transcriptome and network analyses

Caner Karaca[1+], Ezgi Demir Karaman[2+], Asım Leblebici[1], Hasan Kurter[1], Hulya Ellidokuz[3], Altug Koc[1], Ender Berat Ellidokuz[4], Zerrin Isik[2*] and Yasemin Basbinar[5*]

[1] Department of Translational Oncology, Institute of Health Sciences, Dokuz Eylul University, Izmir, Turkey

[2] Department of Computer Engineering, Faculty of Engineering, Dokuz Eylul University, Izmir, Turkey

[3] Department of Preventive Oncology, Institute of Oncology, Dokuz Eylul University, Izmir, Turkey

[4] Department of Gastroenterology, Faculty of Medicine, Dokuz Eylul University, Izmir, Turkey

[5] Department of Translational Oncology, Institute of Oncology, Dokuz Eylul University, Izmir, Turkey

[*] zerrin@cs.deu.edu.tr , ybaskin65@gmail.com

[+] these authors contributed equally to this work

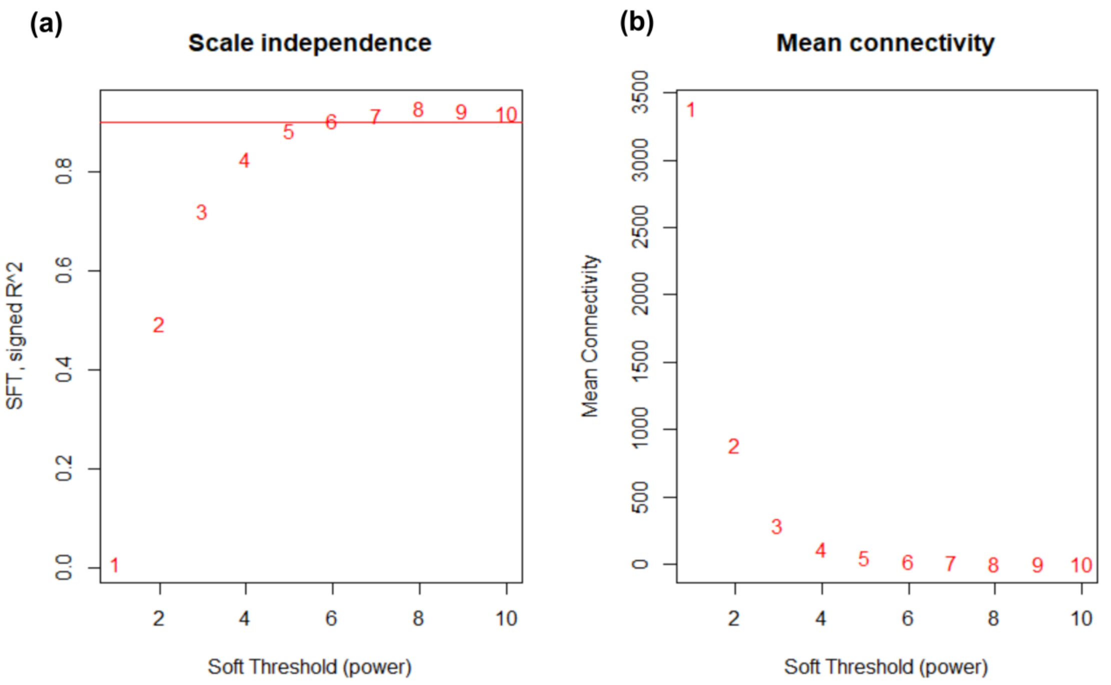

Supplementary Figure S1. Determination of soft-thresholding power in the WGCNA. **(a)** Analysis of the scale-free fit index for various soft-thresholding powers (β). **(b)** Analysis of the mean connectivity for various soft-thresholding powers.

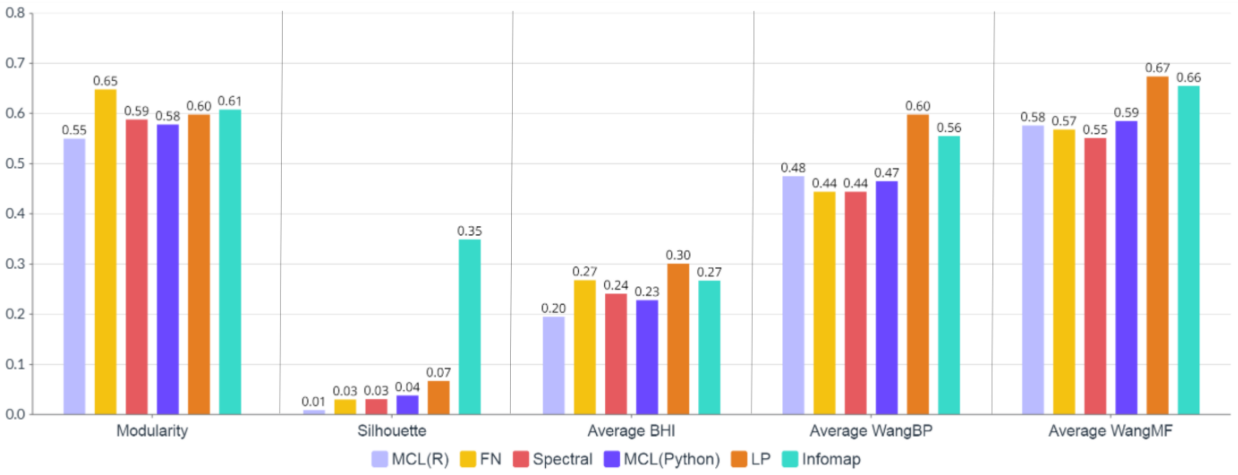

Supplementary Figure S2. Performance comparison of clustering algorithms (Markov clustering (MCL), Fuzzy neighborhood (FN), Spectral, Label Propagation (LP),Infomap) applied for the phenotype of metastasis developed from primary colon cancer.

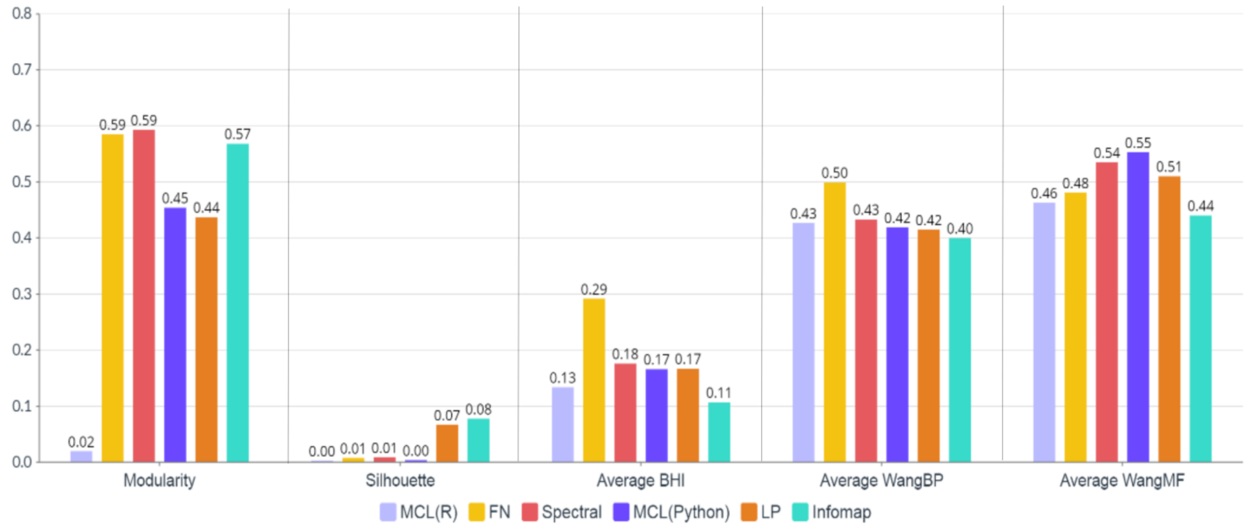

Supplementary Figure S3. Performance comparison of clustering algorithms (Markov clustering (MCL), Fuzzy neighborhood (FN), Spectral, Label Propagation (LP),Infomap) applied for the phenotype of primary colon cancer developed from normal samples.

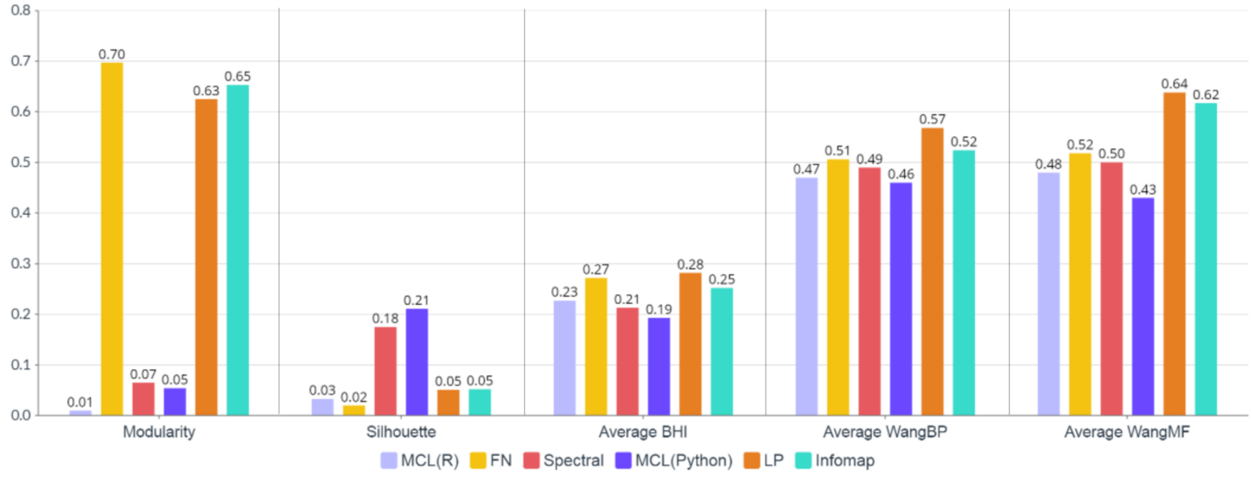

Supplementary Figure S4. Performance comparison of clustering algorithms (Markov clustering (MCL), Fuzzy neighborhood (FN), Spectral, Label Propagation (LP),Infomap) applied for the phenotype of liver metastasis development from normal colon samples.

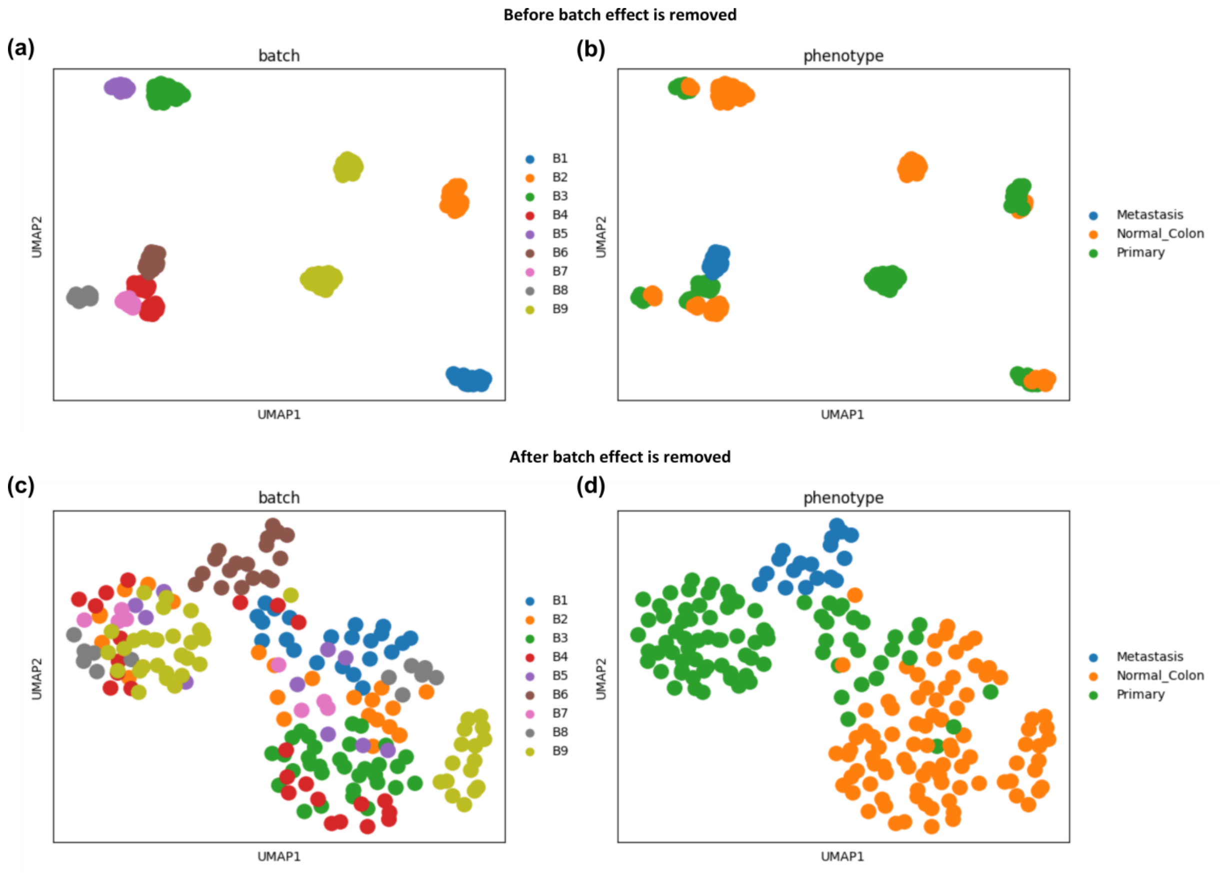

Supplementary Figure S5. UMAP plots of the integrated data before and after batch effect removal **(a)** Nine datasets without batch effect removal, **(b)** Three phenotypes without batch effect removal, **(c)** Nine datasets after batch effect removal, **(d)** Three phenotypes after batch effect removal.

Supplementary Table S1. Significant modules associated with three key phenotypes.

**Module Identifier**	**Main Module Name**	**Associated phenotypes**	**Number of genes**
m2	Metastasis from primary	Development of liver metastases from primary colon cancer	733
m6, m7	Primary from normal	Development of primary colon cancer from normal colon tissue	7537
m16, m17, m18	Metastasis from normal	Development of liver metastases from normal colon tissue	2453

Supplementary Table S2. Gene and interaction numbers in tissue-specific FINs

Module Identifier	Main Module Name	Number of genes in FIN	Number of interactions between the genes in FIN
m2	Metastasis from primary	340	1031
m6, m7	Primary from normal	4353	44265
m16, m17, m18	Metastasis from normal	1347	5301

Supplementary Table S3. Summary of significant submodules detected by the best performing LP and Infomap clustering algorithms for the phenotype of metastasis developed from primary colon cancer.

Clustering Algorithm	Submodule ID	Number of Genes	BHI score	Wang_BP	Wang_MF	Number of downregulated genes	Number of upregulated genes
LP	2	40	0,176	0,342	0,441	0	24
	4	48	0,145	0,302	0,338	0	29
	7	55	0,102	0,257	0,420	1	36
	8	7	0,200	0,499	0,487	1	6
	9	5	0,500	0,734	0,726	0	1
	14	13	0,327	0,434	0,686	0	1
	15	7	0,333	0,594	0,605	1	1
	17	8	0,143	0,394	0,686	0	6
	20	5	0,500	0,651	0,729	0	5
Infomap	1	28	0,340	0,458	0,502	0	20
	2	22	0,318	0,447	0,580	0	16
	3	16	0,321	0,455	0,461	0	14
	7	12	0,326	0,437	0,688	0	1
	8	10	0,500	0,491	0,687	0	9
	12	10	0,255	0,347	0,707	0	5
	13	8	0,500	0,522	0,840	0	8
	15	7	0,381	0,623	0,711	0	7
	16	7	0,333	0,594	0,605	1	1
	19	6	0,300	0,509	0,637	0	1
	25	5	0,500	0,734	0,726	0	1

Supplementary Table S4. Summary of significant submodules detected by the best performing FN and Spectral clustering algorithms for the phenotype of primary colon cancer developed from normal samples.

Clustering Algorithm	Submodule ID	Number of Genes	BHI score	Wang_BP	Wang_MF	Number of downregulated genes	Number of upregulated genes
FN	1	729	0,099	0,273	0,517	0	42
	2	1014	0,204	0,292	0,545	2	83
	7	504	0,100	0,219	0,406	2	19
	8	193	0,412	0,872	0,861	0	2
	43	7	0,500	0,850	0,985	0	0
	51	6	0,500	0,948	0,937	0	1
Spectral	0	1039	0,209	0,287	0,545	2	74
	2	839	0,168	0,266	0,501	4	44
	4	6	0,500	0,948	0,937	0	1
	16	28	0,251	0,398	0,632	1	4
	29	215	0,407	0,836	0,840	0	3
	42	9	0,268	0,420	0,727	0	0
	53	71	0,261	0,418	0,488	2	3

Supplementary Table S5. Summary of significant submodules detected by the best performing FN clustering algorithm for the phenotype of liver metastasis development from normal colon samples.

Clustering Algorithm	Submodule ID	Number of Genes	BHI score	Wang_BP	Wang_MF	Number of downregulated genes	Number of upregulated genes
FN	1	151	0,136	0,315	0,528	19	2
	3	123	0,129	0,281	0,588	14	1
	4	123	0,102	0,274	0,471	25	4
	5	75	0,115	0,281	0,552	28	3
	9	28	0,167	0,349	0,596	2	1
	11	122	0,061	0,267	0,357	15	2
	12	133	0,104	0,269	0,554	35	1
	13	22	0,234	0,446	0,63	3	0
	16	12	0,129	0,37	0,665	2	0
	20	6	0,500	0,636	0,695	2	1

Supplementary Table S7. Significant modules were selected to analyze the occurrence of two phenotypes in the validation dataset

Module Identifier	Main Module Name	Associated phenotypes	Number of genes in the module
m5, m12	Primary from normal	Development of primary colon cancer from normal colon tissue	6504
m14, m15	Primary colon and metastasis from normal	Development of primary colon cancer and metastasis from normal colon tissue	2745

Supplementary Table S8. Common gene counts in significant modules in validation and training datasets.

Module name	Associated phenotypes	Number of Common Genes in both Validation and Training Set
Normal to Primary Colon	Development of primary colon cancer from normal colon tissue	61
Normal to Primary Colon and Metastasis	Primary colon cancer and metastasis from normal colon tissue	42

Supplementary Table S12. Drug screening results obtained for targeting biomarkers in treatment of primary colon cancer.

Target Protein (Biomarker)	Compound Name	Drug Mode of Action	Web Resource
CDKN3	AT-7519	Inhibitor	Chembl Interactions
CDKN3	AZD-5438	Inhibitor	Chembl Interactions
CDKN3	PHA-793887	Inhibitor	Chembl Interactions
CDKN3	RONICICLIB	Inhibitor	Chembl Interactions
EZH2	Lirametostat	Inhibitor	GuideToPharmacology, TTD
EZH2	PF-06821497	Inhibitor	GuideToPharmacology
EZH2	TAZEMETOSTAT	Inhibitor	JAX-CKB, GuideToPharmacology, TTD, OncoKB
NEK2	GSK-579289A	Inhibitor	GuideToPharmacology
NEK2	HESPERADIN	Inhibitor	DTC
TTK	Empesertib	Inhibitor	Chembl Interactions, GuideToPharmacology
TTK	BAY-1217389	Inhibitor	Chembl Interactions
TTK	HESPERADIN	Inhibitor	DTC

Supplementary Table S13. Drug information targeting biomarkers for treatment of liver metastatic colon cancer.

**Target Protein (Biomarker)**	**Compound Name**	**Drug Mode of Action**	**Resource**
IL10RB	PEGINTERFERON λ-1A	agonist	Chembl Interactions
